# Plasma Lycopene Is Associated with Pizza and Pasta Consumption in Middle-Aged and Older African American and White Adults in the Southeastern USA in a Cross-Sectional Study

**DOI:** 10.1371/journal.pone.0161918

**Published:** 2016-09-01

**Authors:** Yuan E. Zhou, Maciej S. Buchowski, Jianguo Liu, David G. Schlundt, Flora A. M. Ukoli, William J. Blot, Margaret K. Hargreaves

**Affiliations:** 1 Department of Internal Medicine, Meharry Medical College, Nashville, TN, 37208, United States of America; 2 Department of Medicine, Vanderbilt University, Nashville, TN, 37212, United States of America; 3 Department of Psychology, Vanderbilt University, Nashville, TN, 37240, United States of America; 4 Department of Surgery, Meharry Medical College, Nashville, TN, 37208, United States of America; University of Kentucky, UNITED STATES

## Abstract

**Background:**

The role of dietary lycopene in chronic disease prevention is not well known.

**Methods:**

This study examined intake of lycopene and other antioxidants from lycopene-rich foods (e.g., pizza and pasta) simultaneously with plasma levels of lycopene and other antioxidants in a representative cross-sectional sample (187 Blacks, 182 Whites, 40–79 years old) from the Southern Community Cohort Study (SCCS). The SCCS is an ongoing study conducted in populations at high risk for chronic diseases living in Southeastern United States. Dietary intake was assessed using a validated food frequency questionnaire (FFQ), and plasma levels of lycopene and other antioxidants were measured at baseline (2002–2005). The participants were classified into tertiles according to consumption of pizza and pasta food groups.

**Results:**

Lycopene dietary intake and plasma lycopene concentrations were significantly higher in the highest (tertile 3) compared to tertiles 1 and 2 (both P < 0.01). Total energy intake ranged from 1964.3 ± 117.1 kcal/day (tertile 1) to 3277.7 ± 115.8 kcal/day (tertile 3) (P<0.0001). After adjusting for age and energy intake, total dietary fat, saturated fatty acids, *trans*-fatty acids, and sodium intakes were significantly higher in tertile 3 than tertiles 2 and 1 (all P <0.01). Vitamin C intake was significantly lower in tertile 3 than tertiles 1 and 2 (P = 0.003). Except for γ-tocopherol being higher in tertile 3 than tertiles 1 and 2 (P = 0.015), the plasma concentrations of antioxidants were lower in tertile 3 than tertiles 1 and 2 (β-carotene, α-carotene, lutein and zeaxanthin, all P<0.05).

**Conclusions:**

In the SCCS population, pizza and pasta were the main sources of dietary lycopene and their intake was associated with plasma lycopene concentration. Diets with frequent pizza and pasta consumption were high in energy, saturated fatty acids, *trans*-fatty acids, sodium and low in other antioxidants. Future studies of lycopene as a protective dietary factor against chronic disease should consider the overall nutritional quality of lycopene-containing foods.

## Introduction

Lycopene is one of the major plant food carotenoids [1]. It is a potent antioxidant with the strongest single oxygen quenching capacity among the carotenoids *in vitro* [2] and it is believed to mitigate oxidative stress effects on cells *in vivo* [3]. In a randomized clinical trial, a lycopene-depleted diet resulted in increased oxidative stress measures [4]. Lycopene is potentially protective against chronic diseases, including cancer, cardiovascular disease, type 2 diabetes, and osteoporosis [5]. However, lycopene’s role in chronic disease prevention has been inconclusive [6, 7].

In the United States, tomatoes and tomato-based foods are the major dietary sources of lycopene [8]. Other lycopene food sources listed in the United States Department of Agriculture (USDA) nutrient database include pasta (e.g., ravioli, spaghetti, lasagna), pizza with meat and vegetable toppings, soups, meat sauces, chili with beans, burritos, hamburgers, watermelon, grapefruit, and papayas [8]. Unlike other antioxidants, lycopene’s bioavailability is improved by food processing, including mechanical or heat disruption of the plant cells and extraction of lycopene into the lipophilic phase in commonly used products such as tomato sauces and ketchup [9, 10]. Thus, a high intake of lycopene may not indicate the high consumption of fresh vegetables and fruits.

In our preliminary analyses of the National Health and Nutrition Examination Survey (NHANES) data from the southern US regions using US Department of Agriculture (USDA) food composition data [8], pizza and spaghetti with meat/tomato sauce were the top two food sources of dietary lycopene (spaghetti contributed an average of 13,501 μg lycopene per day, followed by pizza which contributed an average of 6,956 μg lycopene per day). Also, NHANES 1999–2000 reported pizza and macaroni and cheese among major sources of energy in the American diet [11]. Although there have been previous studies that examined determinants of lycopene status’ including diet, anthropometric and lifestyle factors [12–16], to the best of our knowledge, the associations of nutrient intakes from lycopene-rich diets with simultaneously measured plasma antioxidant concentrations in people consuming such diets have not been reported previously.

We hypothesized that diets containing a high lycopene level would contain also high amounts of energy, total fat (TF), saturated fatty acids (SFA), *trans*-fatty acids (*trans*-FA), and sodium. Dietary intake and plasma antioxidant measures were collected from the SCCS participants [17]. We previously reported that plasma concentrations of lycopene were correlated with estimated dietary intakes as part of the SCCS dietary questionnaire validation efforts [18].

The objective of the present study was to assess intakes of energy, macronutrients, lycopene and other selected micronutrients and examine their potential associations with plasma levels of lycopene and other carotenoids in a representative sample of the SCCS participants. We expected that such information would contribute to knowledge about lycopene’s putative role in disease prevention.

## Materials and Methods

### Ethics Statement

The study was conducted according to guidelines laid down in the Declaration of Helsinki and all procedures involving human subjects were approved by the Institutional Review Boards at Meharry Medical College and Vanderbilt University. Written informed consent was obtained from all subjects.

### Study Population

The SCCS is a prospective cohort study investigating health disparities among predominantly low-income Blacks and Whites across 12 southeastern states. Details have been published previously [17] and can be found on the SCCS website: http://www.southerncommunitystudy.org/. At participating community health centers (CHCs), a computer-assisted interview was conducted to collect information from consented participants.

### Assessment of Dietary Intake

The dietary intakes were ascertained using a validated 89-food items food frequency questionnaire (SCCS FFQ) [19], which was developed to capture the food items commonly consumed by the SCCS population [20]. Among the 89-food items, three were for pasta and pizza (pizza including those homemade, frozen, or from a restaurant; spaghetti including ravioli, lasagna, or other pasta with tomato or meat sauce; macaroni and cheese), nine were specific for fruits and fruit juices, 13 for vegetables, seven for rice, beans, and potatoes, and 16 for meat and meat containing meals.

Mean daily intake of a nutrient contributed by a food item was computed as the product of daily intake frequency of this food, its estimated portion size, and the content of the estimated nutrient in this food. The SCCS study calculated the nutrient composition of each SCCS FFQ food item and its portion size consumed, based on the gender, race and region-specific 24-hour dietary recall data in the NHANES (2003–2004, 2001–2002, 1999–2000, 1994–1996, 1988–1994) and the Continuing Survey of Food Intakes by Individuals (CSFII) (1994–1996) [19]. More specifically, these NHANES and CSFII data were obtained from the 30–84 year old participants (n = 23,398) living in the southern USA, who were either Non-Hispanic white or Non-Hispanic black [19]. For example, the nutrient content of pizza consumed by a Black male SCCS participant was calculated based on the average nutrient content of all pizza consumption reported by Black males in the south and who participated in the NHANES and CSFII databases [19]. The portion size was estimated as the average portion size of pizza consumed by all Black men in these databases. In the SCCS questionnaire, there were nine food frequency categories from “never”, “rarely”, “1/month”, “2-3/month”, “1/week”, “2-3/week”, “4-6/week”, “1/day”, to “2 or more times per day”, which were converted into the daily consumption frequencies of the reported dietary intake. The mean daily dietary intake of a nutrient consumed per day was the sum of this nutrient contributed by all the food items from the SCCS FFQ.

### Sample Selection and Measurement of Plasma Antioxidants

Baseline blood specimens were provided by 12,162 participants who were recruited between March 2002 and October 2004 [18]. Plasma preparation and storage has been described elsewhere [17], with blood donors and non-donors similar on age, gender, and socioeconomic characteristics. Both FFQ information and non-fasting venous blood samples were collected from individuals selected through stratified randomization. In brief, a 2×2×3×3 factorial design with 11 persons within 36 strata ensured balance by race (black/white), sex (male/female), smoking (never/former/current), and body mass index (BMI, kg/m^2^) [21]. Blood samples had been stored up to three years (median 1.6 years) and had not been thawed previously. Plasma carotenoid concentration was quantitated by high-pressure liquid chromatography (HPLC) with photo array detection (DAD), which analysis procedure was detailed previously [22, 23]. The assay calibration and sample handling have been described previously [24, 25]. In this study, trans-lycopene was analyzed since it is the dominant type of isomer in primary lycopene food sources such as tomatoes and tomato products [26]. For quality control, plasma and serum standard references level of each analyte was routinely measured, with the coefficient of variation less than 10% for both the analytes and control pools. For the carotenoids, intraclass correlation coefficients were from 0.73 to 0.93 [18].

### Statistical Analyses

Among the 373 individuals with plasma lycopene measures available, those excluded (n = 4) had missing dietary intake information, had an estimated total energy intake outside the allowable range (600–8,000 kcal/day), or were missing laboratory measures of all antioxidants. Because no significant interaction of race with associations tested was found, the remaining 369 individuals were pooled together and stratified into tertiles by the frequency of pizza and pasta consumption. This stratification was based on our previous nutrient analyses on the diets of the NHANES and CSFII participants, in which pasta and pizza contributed nearly half of the dietary lycopene (data not shown). Socio-demographic and lifestyle measures across the tertiles were compared using ANOVA for continuous and chi-square for discrete data. Daily dietary fat intake was assessed using the Dietary Reference Intakes (DRI) for macronutrients and the Acceptable Macronutrient Distribution Range (AMDR), which recommends 20% to 35% of total energy intake from fat for adults (19 years old and older) [27]. Daily sodium intake was evaluated based on the DRI for sodium, which recommends the daily sodium intake for the general adult population (19 years old and older) below the Tolerable Upper Intake Levels (UL) (2300 mg/day) [28]. Given the concern for excessive dietary fat and sodium consumption, the percentage of individuals with fat intake above 35% and sodium intake above 2300 mg/day was calculated. The percentage of individuals with dietary fat intake and sodium intake above recommended daily levels across tertiles were compared using chi-square statistics. Differences in dietary intake and plasma concentration of antioxidants in the tertiles were assessed using generalized linear regression models. Total energy intake was adjusted for age and intakes of nutrients were adjusted for age and energy intake. Plasma antioxidants were adjusted for age, total energy intake, and LDL-cholesterol [29]. The ratio of unsaturated fatty acids to saturated fatty acids was computed as the sum of monounsaturated fatty acids and polyunsaturated fatty acids divided by the sum of saturated fatty acids. Associations of food groups consumption frequency with dietary lycopene intake and plasma lycopene concentration were analyzed using Spearman correlations. Continuous variables (i.e. age and frequency of food consumption) are presented expressed as means ± SD. Presented mean ± SE values were adjusted for total energy and nutrient intakes and plasma concentrations of antioxidants. Statistical significance was set at P<0.05. All analyses were performed using SAS software (version 9.3, SAS Institute Inc., Cary, NC).

## Results

### Population Characteristics

#### Socio-Demographic Factors

There were no significant differences by gender, race, education or household income levels across the tertiles of pizza and pasta consumption (Table 1). The average age was slightly lower in tertile 3 (50.6±8.9 years) than tertiles 1 and 2 (53.7±9 years and 53.8±10 years respectively) (P = 0.009). Individuals with less than high school education accounted for 28.5% participants in tertile 3 compared to 37.0% in tertile 1 and 33.9% in tertile 2. Across the tertiles, females accounted for 46.3% to 53.8% and Blacks for 46.5% to 58.8% of participants. The majority of participants (~60%) in all tertiles came from households with annual income less than $15,000.

**Table 1:** Population characteristics of study participants (n = 369) across the pizza and pasta consumption frequency tertiles.

	Pizza pasta consumption frequency			P value^a^
	Tertile 1	Tertile 2	Tertile 3	
	(n = 119)	(n = 127)	(n = 123)	
	0–0.8	0.9–2.1	2.2–28	
**Socio-demographic Factors**				
Age (years)^b^	53.7 ± 9	53.8 ± 10	50.6 ± 8.9	0.009
**Gender (%)**				
Female	53.8	50.4	46.3	ns
Male	46.2	49.6	53.7	
**Race (%)**				
Black	58.8	46.5	47.2	ns
White	41.2	53.5	52.8	
**Education (%)**				
Less than high school	37.0	33.9	28.5	ns
High school and above	63	66.1	71.5	
**Household income/year (%)**				
<$15,000	62.2	61.1	57.4	ns
$15,000-$24,999	17.7	21.4	26.2	
≥$25,000	20.1	17.5	16.4	
**Life styles**				
Smoking (%)				
Never	34.5	29.9	33.3	ns
Former smoker	37.8	33.1	30.9	
Current smoker <1 pack/day	16	17.3	22.8	
Current smoker ≥ 1 pack/day	11.8	19.7	13	
**Body mass index (BMI, kg/m**^**2**^**)**^b^	28.5 ± 5.7	27.6 ± 5.2	28.5 ± 6	ns
**Obesity (BMI ≥ 30) (%)**	35.3	27.6	35	ns
**Multivitamin supplement use (%)**	45.4	47.2	39	ns

^a^ P value is the significance level of the global test of the differences among the tertiles.

^b^ Values are means (±SD).

#### Lifestyles

There were no significant differences across tertiles in smoking behavior, obesity prevalence, and multivitamin supplements usage (Table 1). Current smokers accounted for 27.8%, 37.0%, and 35.8% in tertiles 1 to 3, respectively. Obesity (BMI ≥ 30) prevalence ranged from 35.3%, 27.6%, to 35% in tertiles 1 to 3. Multivitamin supplements use varied from 45.4%, 47.2% to 39.0% from tertile 1 to 3.

### Dietary Intakes

Estimated daily intake of energy, macro and micronutrients are in Table 2. Total energy intake increased significantly as tertiles increases (tertile 1, 1964.3 ±117.1; tertile 2, 2278.4±110.5; tertile 3, 3277.7±115.8; kcal/day) (P<0.0001) (Table 2). Similarly, intakes of protein, total dietary fat, saturated fatty acids (SFA), and *trans*-fatty acids (*trans*-FA) adjusted for age and energy intake were significantly higher in tertiles 2 and 3 than in tertile 1. From tertiles 1 and 2 to tertile 3, SFA intake ranged from 27.4± 0.7 and 27.8 ± 0.7 to 32.1 ± 0.8 g/day, respectively (P<0.0001). The ratio of unsaturated fatty acids (UFA) to SFA intakes was slightly but significantly lower in tertile 3 than tertiles 1 and 2 (P = 0.01). Sodium intake was higher in tertile 3 (4645.1±91.8 mg/day) than in tertiles 1 and 2 (4162.7 ± 90.1 mg/day and 4085.6±83.1 mg/day) (P<0.0001). Potassium intake was similar across the tertiles, but the ratio of potassium to sodium intakes was significantly lower in tertile 3 than tertile 1 and tertile 2 (P = 0.0004).

**Table 2:** Dietary intakes among study participants (n = 369) across the pizza and pasta consumption frequency tertiles^a^.

Dietary intake (daily)	Pizza pasta consumption frequency			P value^b^
	tertile 1	tertile 2	tertile 3	
**Total energy intake (kcal)**^c^	1964.3 ± 117.1	2278.4 ± 110.5	3277.7 ± 115.8	<0.0001
**Macronutrients**^d^				
Protein (g)	92.6 ± 2.4	90.5 ± 2.2	101.2 ± 2.4	0.004
Carbohydrates (g)	304.9 ± 5.6	298 ± 5.1	297.1 ± 5.7	ns
Sugar^e^ (g)	140.7 ± 5.3	136.5 ± 4.9	128.7 ± 5.4	ns
Total fat (g)	92.2 ± 2.2	92.5 ± 2.0	104.1 ± 2.2	0.0002
Saturated fatty acids (g)	27.4 ± 0.7	27.8 ± 0.7	32.1 ± 0.8	<0.0001
unsaturated fat/saturated fat	2.2 ± 0.04	2.1 ± 0.04	2 ± 0.04	0.01
*Trans*-fatty acids^f^ (g)	7.7 ± 0.2	7.6 ± 0.2	8.5 ± 0.2	0.001
Fiber (g)	21.8 ± 0.7	21 ± 0.7	21.2 ± 0.7	ns
**Micronutrients**^d^				
Sodium (mg)	4162.7 ± 90.1	4085.6 ± 83.1	4645.1 ± 91.8	<0.0001
Potassium (mg)	3350.3 ± 67.1	3300.2 ± 61.9	3285.7 ± 68.3	ns
Potassium/Sodium	0.9 ± 0.02	0.8 ± 0.02	0.7 ± 0.02	0.0004
**Antioxidant nutrients**^d^				
Lycopene (μg)	4108.5 ± 303	4983.3 ± 279.3	8375.8 ± 308.6	<0.0001
β-carotene (μg)	4544.9 ± 328.7	4602.9 ± 303	4187.6 ± 334.7	ns
α-carotene (μg)	532.6 ± 48	548.7 ± 44.2	503.9 ± 48.8	ns
Lutein and Zeaxanthin (μg)	3934.8 ± 337	3866.5 ± 310.6	2943 ± 343.2	ns
β-cryptoxanthin (μg)	265.4 ± 18.3	236.8 ± 16.8	173.9 ± 18.6	0.003
Vitamin C (mg)	148.1 ± 7.6	136.1 ± 7	109.9 ± 7.7	0.003
α-tocopherol (mg)	8.5 ± 0.2	8.3 ± 0.2	9 ± 0.2	ns

^a^ Tertiles 1, 2 and 3 include the individuals consuming pizza/pasta 0 to 0.8 times/week, 0.9 to 2.1 times/week, and >2.2 times per week respectively.

^b^ P value is the significance level of the global test of the differences among the tertiles.

^c^ Values are adjusted means (±SE); adjusted for age.

^d^ Values are adjusted means (±SE); adjusted for age and total energy intake.

^e^ Sugar was included in CHO intake.

^f^ *trans*-fatty acids content was calculated by subtracting a sum of saturated fatty acids and unsaturated fatty acids (monounsaturated and polyunsaturated fatty acids) from total fatty acids mean.

Estimated daily intake of dietary lycopene was significantly higher (P<0.0001) in tertile 3 than in tertiles 2 and 1 (8,375±309, 4,983±279, and 4,109±303 μg/day, respectively). Intakes of other antioxidant micronutrients did not vary significantly across tertiles (α- and β- carotene, α-tocopherol) or were lower (β-cryptoxanthin) with higher consumption of pizza and pasta. Vitamin C was significantly lower as the tertiles increased (tertile 1, 148.1±7.6; tertile 2, 136.1±7; tertile 3, 109.9±7.7; mg/day) (P = 0.003).

### Dietary intake guidelines

**Fig 1:**
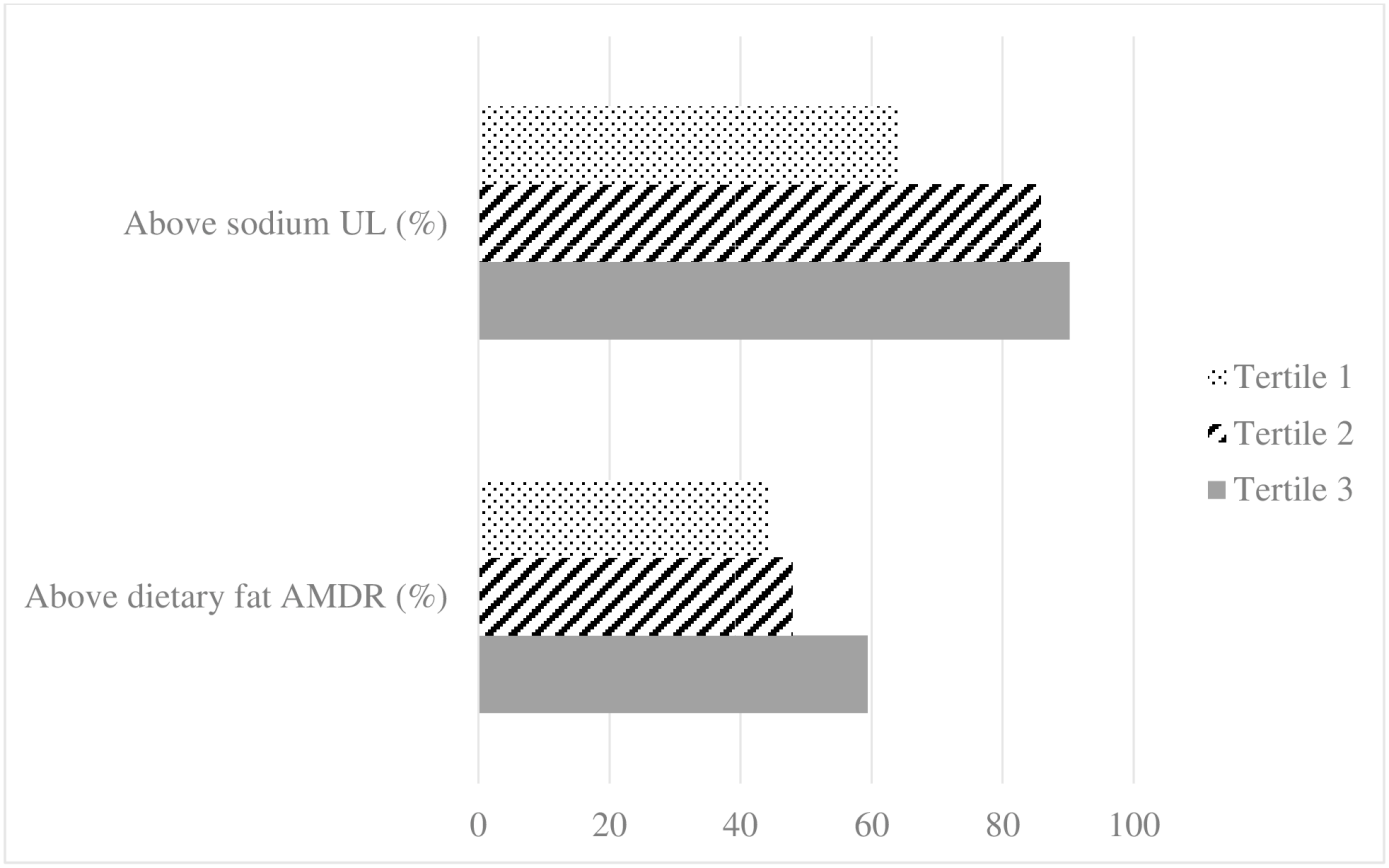
The percentages of individuals with above recommended levels of dietary fat and sodium across tertiles^a^. ^**a**^ Daily dietary fat beyond the Acceptable Maconutrient Distribution Range(AMDR)(20%–35%) in tertile 1, tertile 2 and tertile 3, P = 0.054; daily sodium intake beyond the Tolerable Upper Intake Levels (2300 mg/fay) in tertile 1, tertilr 2 and tertile 3, P<0.0001.

Daily dietary fat levels above the recommended 35% upper limit for dietary fat [27] were 44.5%, 48%, and 59.4% in tertile 1, tertile 2 and tertile 3 respectively (P = 0.054) (Fig 1). Daily sodium intake above the recommended 2300 mg/day upper limit [28] were 63.9%, 85.8%, and 90.2% in tertile 1, tertile 2 and tertile 3 respectively (P<0.0001) (Fig 1).

### Plasma Antioxidant Concentrations

Plasma antioxidant concentrations were adjusted for age, total energy intake, and total-cholesterol level. In the present study, total-cholesterol showed the strongest correlation with plasma lycopene (R = 0.322 and P<0.0001) among the cholesterol measurements (HDL-cholesterol and LDL-cholesterol). Plasma lycopene concentration in tertile 1 was lower than in tertile 2 and 3 (28.3±1.3, 34.1±1.2, and 31.9 ±1.4 μg/dL respectively, P = 0.006) (Table 3). Plasma γ-tocopherol in tertile 3 was significantly higher than tertiles 1 and 2. In contrast, plasma concentrations of ß-carotene at tertile 1 (16.8±1.4) μg/dL and tertile 2 (13.3±1.4) μg/dL were significantly higher than tertile 3 (10±1.5) μg/dL (P = 0.002). Plasma α-carotene and lutein/zeaxanthin were significantly lower in tertile 3 compared to tertiles 1 and 2 (Table 3). Similarly, plasma β-cryptoxanthin concentration was lower in tertiles 2 and 3 than tertile 1 of pizza/pasta consumption (Table 3).

**Table 3:** Plasma concentration of antioxidant nutrients across the pizza and pasta consumption frequency tertiles (n = 369)^a^.

Antioxidant concentration in plasma (μg/dl) ^b^	Pizza pasta consumption frequency			P value^c^
	tertile 1	tertile 2	tertile 3	
lycopene	28.3 ± 1.3	34.1 ± 1.2	31.9 ± 1.4	0.006
β-carotene	16.8 ± 1.4	13.3 ± 1.4	10 ± 1.5	0.008
α-carotene	3.4 ± 0.3	3.1 ± 0.3	2.2 ± 0.3	0.024
α-tocopherol	1286 ± 57	1267 ± 53	1202 ± 58	ns
γ-tocopherol	300 ± 14	296 ± 13	348 ± 14	0.015
lutein and zeaxanthin	19.2 ± 0.9	17.5 ± 0.8	15.1 ± 0.9	0.008
β-cryptoxanthin	8 ± 0.5	7.8 ± 0.5	6.4 ± 0.5	ns

^a^ Tertiles 1, 2 and 3 include the individuals consuming pizza/pasta 0 to 0.8 times/week, 0.9 to 2.1 times/week, and >2.2 times per week, respectively.

^b^ Values are adjusted means (±SE); adjusted for age, total energy intake, and total-cholesterol plasma concentration.

^c^ P value is the significance level of the global test of the differences among the tertiles.

### Association of Dietary Lycopene Intake with Plasma Lycopene Concentrations

Dietary lycopene intake, adjusted for age and energy intake, showed the strongest correlation with the frequency of pizza and pasta consumption (R = 0.54, P<0.0001) (Table 4). Pizza and pasta but no other foods were significantly correlated with plasma lycopene adjusted for age and total cholesterol (R = 0.14, P = 0.009) (Table 4).

**Table 4:** Correlation^a^ of food group consumption frequency with dietary lycopene intake and plasma lycopene concentrations (n = 369).

Food group (times/week)	Consumption frequency	Dietary lycopene intake (μg/day)		Plasma lycopene (μg/dL)	
	Mean^b^	R _spearman_^c^	P	R _spearman_^d^	P
Pasta and pizza	2.3 ± 3.1	0.54	<0.0001	0.14	0.009
Vegetables	17.2 ± 12.9	0.29	<0.0001	0.05	ns
Rice and legumes	6.2 ± 5.5	0.25	<0.0001	0.07	ns
Meat	8 ± 8.1	0.25	<0.0001	-0.08	ns
Fruits	13.6 ± 13.5	0.14	0.006	0.01	ns

^a^ Values are Spearman correlation coefficients.

^b^ Values are means (±SD).

^c^ Values are adjusted for age and total energy intake.

^d^ Values are adjusted for age, total energy intake, and total-cholesterol plasma concentration.

## Discussion

A major finding of this study is that in the SCCS cohort, pizza and pasta were the main sources of dietary lycopene, and their consumption was positively associated with plasma lycopene concentration. Diets of individuals in the highest tertile of pizza and pasta intake contained significantly higher amounts of energy, SFA, *trans*-FA, and sodium, and low β-cryptoxanthin and vitamin C compared to those in the lowest tertile. Also, in individuals with high plasma lycopene concentration, their plasma concentrations of *α*- and *β*- carotene, lutein and zeaxanthin were significantly lower.

Traditionally, the Southeastern US population is known for consuming deep fried foods, organ meats, and excessively boiled vegetables [30, 31]. The US National Health and Nutrition Survey (NHANES) have documented that the southeastern population reported the highest intake of sodium, but the lowest consumption of fiber, potassium, calcium, iron, magnesium, and vitamins A and C from all US regions [32]. More recently, the REGARDS study conducted in eight southeastern US states revealed five common dietary patterns termed “convenience” (Chinese and Mexican foods, pizza, and other mixed dishes), “plant-based” (fruits and vegetables), “sweets/fats” (sugary foods), “southern” (fried foods, organ meats, and sweetened beverages), and “alcohol/salads” (alcohol, green-leafy vegetables, and salad dressing) [33, 34]. Another study among 1904 Blacks and Whites in North Carolina found several dietary patterns via principal component analysis, including high fat/meat/potatoes, vegetable/fish/poultry and fruit/whole grain [35]. In the present study, the high frequency of pizza and pasta consumption may reflect a “convenience” dietary pattern, which is marked by high dietary fat, particularly SFA, and high sodium intake. For example, the proportions of individuals above the AMDR (20%-35%) for dietary fat and above the UL (2300 mg/day) for sodium intakes were higher in tertile 3 than tertiles 1 and 2 (S1 Table). Also, we noted that age and total energy adjusted fruit consumption was higher in lower pizza/pasta tertiles (15.2 ± 1.1, 13.9 ± 1.0, 10.3 ± 1.1 times/week, P = 0.007; S2 Table). In the current study, vegetable and fruit consumption were significantly correlated with intakes of ß-carotene and α-carotene but not lycopene (S3 and S4 Tables). The observed food and nutrient intake patterns across the pizza/pasta tertiles were consistent with the US-wide CSF II study [36]. In that study, fast food consumers reported higher consumption of meat and starch mixed dishes such as pizza and lasagna, higher intake of total energy, fat, cholesterol, sodium, and calcium, but lower intake of protein, dietary fiber, vitamins A and vitamin C, and β- carotene compared to the not fast food consumers. In addition to the NHANES 1999–2000 report [11], a study assessing the diet of African American women also showed that pizza and macaroni and cheese were the main sources of energy [37]. Furthermore, it has been reported that among women with low-socioeconomic status in the US, the consumption of high fat and starch-based mixed dishes had increased in the past few decades [38]. Additionally, a previous study in African American adults reported that snack chips were the primary food source of α- and γ- tocopherol [39]. In the present study, plasma γ-tocopherol concentrations were higher within higher tertiles of pizza and pasta intake. Results from the current study adds additional evidence showing that mixed dishes and convenience foods are important sources of lycopene and other antioxidants in the American diet.

It has been documented that the southeastern US population has a higher prevalence of hypertension [40] and related co-morbidities, including end-stage renal disease [41], cardiovascular diseases [42], and type 2 diabetes [42], than other US regions. Etiology of these chronic diseases is attributed, at least in part, to oxidative stress and chronic inflammation [43]. In addition, excessive energy intake leads to obesity which is now regarded as a condition of chronic low-grade inflammation [43]. Antioxidant nutrients can attenuate the oxidative damage and enhance the antioxidant defense against inflammation [44, 45]. Dietary SFA and *trans*-FA are suggested to endorse pro-inflammation status that can result in cardio-metabolic risk factors such as hyperlipidemia and impaired vascular function [46, 47]. High sodium but low potassium intakes have been long considered a factor in the etiology of hypertension among individuals genetically susceptible to essential hypertension [48]. High sodium consumption could also contribute to the development of cardiovascular diseases especially stroke [49]. For example, results from the REGARDS study, a national cohort on stroke, showed that high sodium intake was associated with increased risk for kidney damage [50] and high *trans*-FA intake was related to increased risk of stroke [51]. Hypertension, a major preventable risk factor associated with mortality and disability-adjusted life years [49], lower health-related quality of life [52] and a higher probability of premature mortality [53], was reported more frequently in the Southeast compared to other US regions.

A major limitation of our study is the possibility of under-reporting or over-reporting that is common in studies using self-reported FFQ to assess energy and nutrient intakes [54–56]. Also, an FFQ that includes a comprehensive list of mixed dishes with tomato-based ingredients might have more accurately captured dietary lycopene intake than the SCCS FFQ we used. However, in our validation study, the plasma concentrations of major carotenoids and tocopherol showed significant positive associations with the FFQ estimated intakes [19]. It has been suggested that because of the conversion from trans-lycopene to cis-lycopene in humans [57], the two isomers of lycopene in plasma should be detected separately. However, in this study, we only measured trans-lycopene plasma concentration.

It is possible that the observed differences in dietary intake and plasma concentrations of antioxidants across pizza and pasta consumption tertiles might have been driven by other confounding factors not included in the analyses. However, we have examined several important socio-demographic and lifestyle variables including age, gender, race, income, education, smoking, obesity and multivitamin usage, which showed no association with dietary intake and plasma measures. Finally, the relatively small sample size restricted performing more advanced analyses to explore potential associations of antioxidants’ plasma concentration with food clusters reflective of different eating patterns.

In this study we found a moderately positive association between dietary lycopene and plasma lycopene. We were able to find two studies that also used FFQs for assessing the association of dietary and plasma lycopene. One study was conducted in a predominantly non-Hispanic Whites population from the US states of Connecticut and Florida and reported rather strong correlations between dietary lycopene and plasma lycopene (R = 0.29, P = 0.002) [58]. In contrast, a study among low-income African Americans in the state of Georgia showed no associations between dietary lycopene and serum lycopene [59]. There are most likely numerous reasons for discrepancies between these studies and our study including different methodology, and populations studied. Future clinical and epidemiological studies designed to explore further the observed associations of pizza and pasta consumption with lycopene intake and plasma concentrations of lycopene and other antioxidants in various populations are warranted.

## Conclusions

The present study demonstrated that pizza and pasta were the main sources of dietary lycopene among predominantly low-income middle-aged and older Blacks and Whites living in the southeastern US. The dietary pattern characterized by frequent pizza and pasta consumption (>2 times per week) was associated with a higher lycopene intake and higher lycopene plasma concentration, but it was also high in energy, SFA, *trans*-FA and sodium intakes. Thus, a diet with a frequent pizza and pasta intake presents a nutrient profile inconsistent with dietary recommendations that aim to reduce chronic disease risk in middle-aged and older adults at high risk of chronic diseases living in the Southeastern USA. Future studies of lycopene as a protective dietary factor against chronic disease should consider the overall nutritional quality of lycopene-containing foods.

## Supporting Information

S1 Table Proportion of dietary fat and sodium consumption above dietary. (DOCX)

S2 Table Food group consumption frequency across the pizza and pasta consumption frequency tertiles (n = 369). (DOCX)

S3 Table Correlation of food group consumption frequency with dietary α-carotene intakes and plasma α-carotene concentrations (n = 369). (DOCX)

S4 Table Correlation of food group consumption frequency with dietary β-carotene intakes and plasma β-carotene concentrations (n = 369). (DOCX)
