# Supplementary material for: Plasma Lycopene Is Associated with Pizza and Pasta Consumption in Middle-Aged and Older African American and White Adults in the Southeastern USA in a Cross-Sectional Study
Source: PLoS One. 2016 Sep 1;11(9):e0161918. doi: 10.1371/journal.pone.0161918 (PMC5008825; doi:10.1371/journal.pone.0161918)
Supplement: S1 Table — (DOCX) [file pone.0161918.s001.docx]

**Appendix**

**S1 Table Proportion of dietary fat and sodium consumption above dietary recommendations across the pizza and pasta consumption frequency tertiles (n=369)^a^**

|  | **Pizza pasta consumption frequency** | | | | | | **P value^b^** |
| --- | --- | --- | --- | --- | --- | --- | --- |
|  | tertile 1 | | tertile 2 | | tertile 3 | |  |
| Dietary fat (>35% energy)^c^ (%) | 44.5 |  | 48 |  | 59.4 |  | 0.054 |
| Sodium (>2300 mg/day)^d^ (%) | 63.9 |  | 85.8 |  | 90.2 |  | <0.0001 |
| ^a^ Tertiles 1, 2 and 3 include the individuals consuming pizza/pasta 0 to 0.8 times/week, 0.9 to 2.1 times/week, and >2.2 times per week respectively. | | | | | | | |
| ^b^ P value is the significance level of the chi square tests of the proportions among the tertiles. | | | | | | | |
| ^c^ Above the Acceptable Macronutrient Distribution Range (AMDR) of dietary fat (20%~35%) | | | | | | | |
| ^d^ Above the Upper Level of Intake (UL) of dietary sodium (2300 mg/day) | | | | | | | |
