# Supplementary material for: Plasma Lycopene Is Associated with Pizza and Pasta Consumption in Middle-Aged and Older African American and White Adults in the Southeastern USA in a Cross-Sectional Study
Source: PLoS One. 2016 Sep 1;11(9):e0161918. doi: 10.1371/journal.pone.0161918 (PMC5008825; doi:10.1371/journal.pone.0161918)
Supplement: S2 Table — (DOCX) [file pone.0161918.s002.docx]

**Appendix**

**S2 Table Food group consumption frequency across the pizza and pasta consumption frequency tertiles (n=369)^a^**

| **Food group^b^** | **Pizza pasta consumption frequency** | | | | | | **P value^c^** |
| --- | --- | --- | --- | --- | --- | --- | --- |
|  | tertile 1 | | tertile 2 | | tertile 3 | |  |
| Pasta and pizza (times/week) | 0.7 | ± 0.2 | 1.5 | ± 0.2 | 4.4 | ± 0.2 | <0.0001 |
| Vegetables (times/week) | 16.9 | ± 1.1 | 17.9 | ± 1 | 16.7 | ± 1.1 | ns |
| Rice and legumes (times/week) | 6.2 | ± 0.4 | 5.6 | ± 0.4 | 6.1 | ± 0.4 | ns |
| Meat (times/week) | 7.3 | ± 0.5 | 6.8 | ± 0.4 | 8.3 | ± 0.5 | ns |
| Fruits (times/week) | 15.2 | ± 1.1 | 13.9 | ± 1 | 10.3 | ± 1.1 | 0.007 |
| ^a^ Tertiles 1, 2 and 3 include the individuals consuming pizza/pasta 0 to 0.8 times/week, 0.9 to 2.1 times/week, and >2.2 times per week respectively. | | | | | | | |
| ^b^ Values are adjusted means (±SE); adjusted for age, total energy intake. | | | | | | | |
| ^c^ P value is the significance level of the global test of the differences among the tertiles. | | | | | | | |
