# Supplementary material for: Plasma Lycopene Is Associated with Pizza and Pasta Consumption in Middle-Aged and Older African American and White Adults in the Southeastern USA in a Cross-Sectional Study
Source: PLoS One. 2016 Sep 1;11(9):e0161918. doi: 10.1371/journal.pone.0161918 (PMC5008825; doi:10.1371/journal.pone.0161918)
Supplement: S3 Table — (DOCX) [file pone.0161918.s003.docx]

**Appendix**

**S3 Table Correlation of food group consumption frequency with dietary α-carotene intakes and plasma α-carotene concentrations (n=369)^a^**

| **Food group (times/week)** |  | **Consumption frequency** | |  | **Dietary α-carotene intake (µg/day)** | |  | **Plasma α-carotene (µg/dL)** | |
| --- | --- | --- | --- | --- | --- | --- | --- | --- | --- |
|  |  | **Mean^b^** | |  | **R _spearman_^c^** | **P** |  | **R _spearman_^d^** | **P** |
| Pasta and pizza |  | 2.3 | ± 3.1 |  | -0.01 | ns |  | -0.1 | 0.06 |
| Vegetables |  | 17.2 | ± 12.9 |  | 0.68 | <0.0001 |  | 0.24 | <0.0001 |
| Rice and legumes |  | 6.2 | ± 5.5 |  | 0.37 | <0.0001 |  | 0.08 | ns |
| Meat |  | 8 | ± 8.1 |  | 0.33 | <0.0001 |  | 0.1 | 0.07 |
| Fruits |  | 13.6 | ± 13.5 |  | 0.33 | <0.0001 |  | 0.26 | <0.0001 |

^a^ Values are Spearman correlation coefficients.

^b^ Values are means (±SD).

^c^ Values are adjusted for age and total energy intake.

^d^ Values are adjusted for age and total energy intake, and total-cholesterol plasma concentration.
